# The complex pathophysiology of bone fragility in obesity and type 2 diabetes mellitus: therapeutic targets to promote osteogenesis

**DOI:** 10.3389/fendo.2023.1168687

**Published:** 2023-07-20

**Authors:** Siresha Bathina, Reina Armamento-Villareal

**Affiliations:** ^1^ Division of Endocrinology Diabetes and Metabolism, Baylor College of Medicine, Houston, TX, United States; ^2^ Center for Translational Research on Inflammatory Disease, Michael E. DeBakey Veterans Affairs (VA) Medical Center, Houston, TX, United States

**Keywords:** obesity, diabetes, adipogenesis, myogenesis, osteogenesis

## Abstract

Fractures associated with Type2 diabetes (T2DM) are major public health concerns in an increasingly obese and aging population. Patients with obesity or T2DM have normal or better than normal bone mineral density but at an increased risk for fractures. Hence it is crucial to understand the pathophysiology and mechanism of how T2DM and obesity result in altered bone physiology leading to increased fracture risk. Although enhanced osteoclast mediated bone resorption has been reported for these patients, the most notable observation among patients with T2DM is the reduction in bone formation from mostly dysfunction in osteoblast differentiation and survival. Studies have shown that obesity and T2DM are associated with increased adipogenesis which is most likely at the expense of reduced osteogenesis and myogenesis considering that adipocytes, osteoblasts, and myoblasts originate from the same progenitor cells. Furthermore, emerging data point to an inter-relationship between bone and metabolic homeostasis suggesting that these physiologic processes could be under the control of common regulatory pathways. Thus, this review aims to explore the complex mechanisms involved in lineage differentiation and their effect on bone pathophysiology in patients with obesity and T2DM along with an examination of potential novel pharmacological targets or a re-evaluation of existing drugs to improve bone homeostasis.

## 1 Introduction

### 1.1 Obesity type 2 diabetes and bone

Obesity is associated with increased risk of T2DM (1), Cardiovascular diseases (2) and Cancer (3). The World Health Organization (WHO) defined overweight as a BMI of 25 to 29.9 kg/m2 and obesity as a BMI greater than or equal to 30 kg/m2 (4). According to new world health Atlas 2022, by 2030, 20% of women and 14% of men and over 1 billion people will be living with obesity globally (https://www.worldobesityday.org) and nearly 1 in 4 adults will have severe obesity with prevalence of more than 25% higher in 25 states in US (5). Obesity may lead to T2DM and by 2035, the global prevalence of T2DM is likely to be 592 million (6). The duo (obesity and T2DM) increases as the population ages. Both conditions are associated with normal or better than normal bone mineral density (BMD) but paradoxically increase in the risk for fractures. Obesity is a risk factor for T2DM such that the bone phenotype in the two conditions likely overlap in a major way. Thus, this review aims to examine, the complicated underlying molecular mechanisms involved in the alteration in lineage differentiation and identify pharmacological targets that redirect cell differentiation from the adipogenic to the osteogenic/myogenic pathways.

### 1.2 Pathophysiology of skeletal fragility in obesity and T2DM

**Figure 1:**
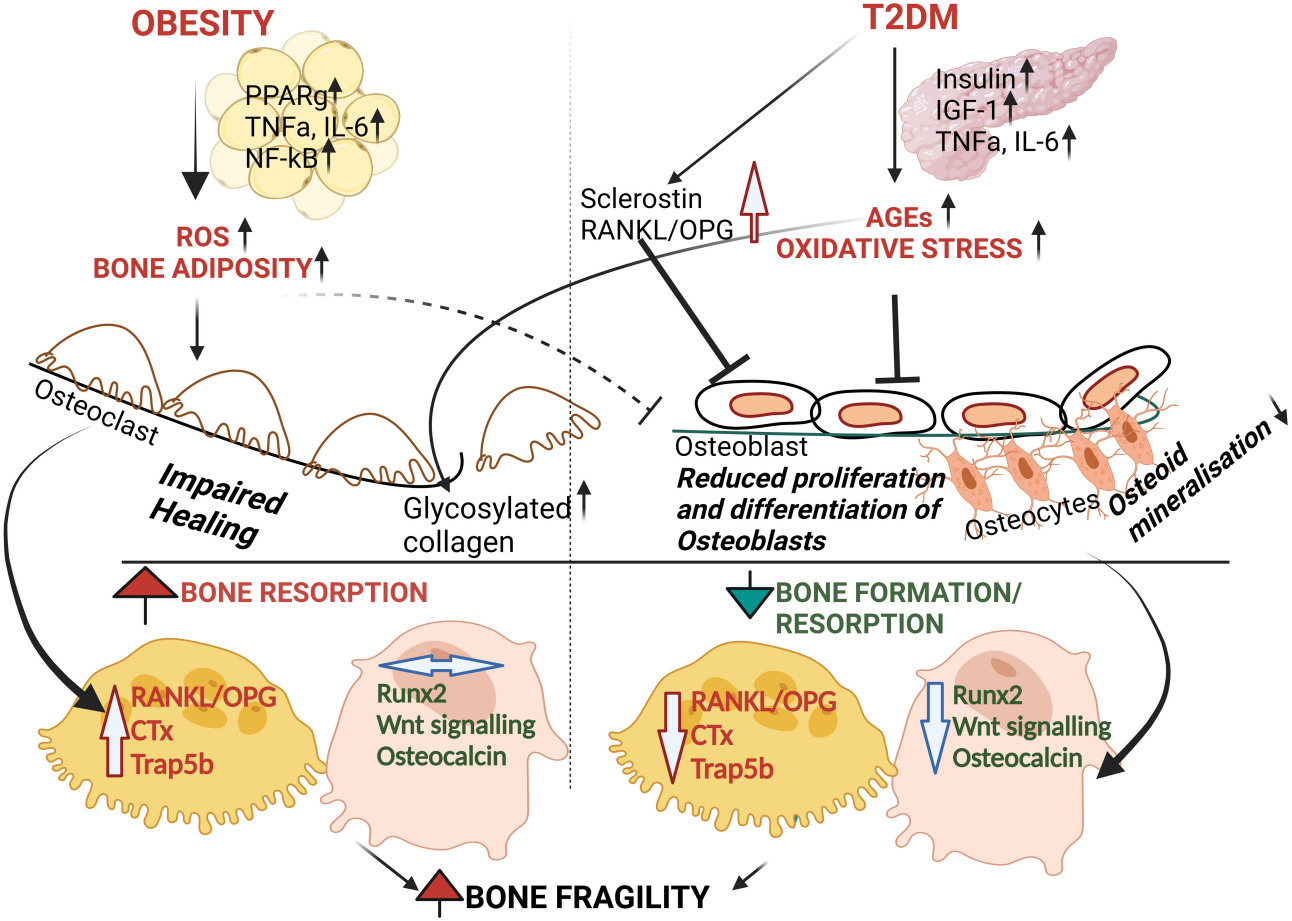
Schematic proposal of pathophysiology of bone fragility in Obesity and T2DM. Even though underlying mechanisms are unclear, both T2DM and Obesity causes oxidative stress and inflammation. T2DM causes the accumulation of AGEs with increased oxidative stress leading to osteoblast dysfunction and reduced bone formation (i.e. reduced Runx2 and OCN) followed by increasing bone fragility. On the other hand, obesity is associated with increased pro-inflammatory markers and pro-resorptive factors such as RANKL, TRAP5b. However, whether obesity by itself has any effect on osteoblastic function is unclear. Altogether, the above contribute to simultaneous effect on bone tissue homeostasis leading to bone fragility^15-21^.

Increase in bone marrow adipose tissue volume has been reported both in diabetes and obesity (7). Earlier studies confirmed an increased risk for hip fracture in both male and female patients with type1 diabetes(T1DM) (8). Osteoporotic fractures especially on the hip, are increased in both T1DM and T2DM, but the risk is 7 fold for those with T1DM compared to 1.38 fold increase in hip fractures of T2DM (9). The increased risk in T1DM is due to lack of anabolic effects of insulin which may contribute to lower peak bone mass while bone mass seems to be preserved in the T2DM (10). Regardless, studies have shown that both T1DM and T2DM is associated with a switch from osteogenesis to adipogenesis, increase in bone marrow adiposity leading to cellular marrow replacement with fat (11). The higher BMD in obesity is believed to be due to skeletal adaptation to accommodate mechanical load and strain (12, 13). However, visceral and total adiposity was not associated with vertebral fractures in men (14). Some studies reported negative correlation between BMD (15, 16). Obesity, is associated with increased secretion of pro-inflammatory factors (as described in **Figure 1**)) that may be harmful to bone and activation peroxisome proliferator-activated receptor-γ (*PPARγ*) and CCAAT/enhancer-binding protein alpha (CEBPa), nuclear factor kappa light chain enhancer of activated B cells (NF-Kb) pathway (17, 18). Adipokines produced in the adipocytes have inverse relationship to fat mass (19, 20), variably effects bone mass (21). Cao et al, found reduced serum bone formation marker osteocalcin (OCN) and increased bone resorption markers, serum C-telopeptide of type I collagen (CTx) and Tartrate-resistant acid phosphatase 5b(TRAP5b) in diet-induced obese mice (22). Furthermore, Jain et al.,studies confirmed that visceral adipose tissue (VAT) is negatively associated with bone mineral density (23). On the other hand, in T2D BMD is normal or above normal,likely protective against vertebral fractures (24), but some studies show reduced BMD (25, 26) due to accumulation of advanced glycation end products (AGEs) (27, 28) increased proinflammatory cytokines such as TNF-a, IL-6 (28, 29) high sclerostin levels (30) leading to reduction in bone formation, OCN (31) and (Procollagen I N-terminal propeptide) P1NP levels in T2DM (31, 32) and impairment in osteoblastogenesis (33–35). There is also reduction in bone resorption markers (CTX and TRAP5b) (28) though bone turnover markers are not as predictive of fractures compared to BMD and maybe difficult to interpret,. Mesenchymal stem cells residents in the bone marrow (BMSCs) are endowed with plasticity and can differentiate into the osteogenic, myogenic or adipogenic lineages depending on the predominant transcription factors present. The enhanced potential of skeletal muscle satellite cells or SMSCs for adipogenic differentiation was observed in diabetic rats using a 3-dimensional matrices *in vitro* model (36) and from in genetically obese Zucker rats (37). Furthermore, myoblasts isolated from Wnt10b (wingless-type mouse mammary tumor virus integration site) null mice showed increased adipogenic potential (38). Jiang et al. found that PRDM16 (Positive Regulatory Domain Motif -16) over expression could partially reverse the effect of mir-499 on adipogenic differentiation of SMSCs and maybe a target for obesity treatment (39). Therefore, there is a need to fully understand the molecular mechanisms behind this shift along with investigations on common regulatory pathways.

Despite the high BMD in obese subjects, these individuals are at increased risk of fractures at nonvertebral skeletal sites (i.e. lower extremities and humerus) (40–42). There are several mechanisms proposed to explain the increased skeletal fragility in obese individuals such as low vitamin D with consequent secondary hyperparathyroidism (43, 44), increased levels of proinflammatory cytokine release from the expanded adipose tissue volume and possibly the high levels of leptin and reduced adiponectin though both have variable effects on the skeleton (44) Low vitamin D is easily corrected clinically but the increase in adipose tissue volume and subsequent proinflammatory state requires more effort (45). Likewise, studies (46–52) have shown that BMD is also higher in patients with T2DM compared to nondiabetic subjects but associated with an increased fracture risk affecting any skeletal site (52, 53). Given that obesity is a risk for T2DM, it would be hard to separate out the effect of obesity from diabetes on the bone. Clinical studies (54–57), including from our group (58, 59) demonstrated suppressed bone formation maker OCN, P1NP (48) and bone resorption marker (CTX) in patients with T2DM. Additionally, Vigevano et al. showed that among obese men, those with concurrent T2DM had higher bone density but reduced bone turnover markers (CTx and OCN) (60) and lower bone strength suggesting that if obesity has a negative effect on the bone, T2DM further adds to the skeletal compromise from obesity alone or that diabetes is the driver for the skeletal phenotype in those who have both. In a study of older women, relative to nonobese without diabetes, those with diabetes but nonobese had a 1.9 risk for vertebral or hip fracture and 1.4 for nonvertebral and non-hip fractures. The corresponding numbers for nondiabetic but obese were 1.2 and 1.1, respectively, while they were 1.5 and 1.8, respectively, for those with both diabetes and obesity (61). Meanwhile, given the clinical observation of increased in bone marrow fat in obesity and diabetes, it is likely that MSCs are involved in the pathology of skeletal fragility seen in in patients with obesity, diabetes or both. This hypothesis was supported by a study from Tencerova et al., which showed that an increase in adipocyte differentiation along with accelerated senescence in BMSCs lead to bone fragility in obese men (62). Thus, pathophysiology of brittle bone in both obesity and T2DM may be attributed as due to the mechanisms discussed below.

### 1.3 Lack of regulation of brown fat synthesis and/or enhancement of adipogenesis

Differentiation of fat and its control is regulated by transcriptional cascade which can affect the physiological functioning of white and brown adipocytes (63). Normally, the conversion of pre-adipocytes to mature lipid containing adipocytes is a multi-step complex process regulated by transcription factors which can be altered by inflammatory signaling pathways of obesity (64). Of all transcription factors, (*PPARg and CEBPa*) are the key regulators in driving fat cell differentiation (65, 66). Crucially, *PPARγ* which is the driving factor for adipogenesis needs co-activation by *CEBPa* to promote myogenesis (67–69) Cohen et al. (70) found that knock out of PRDM16 resulted in obesity and severe insulin resistance mice fed a high-fat diet (70). Several pre-clinical experiments have confirmed the association of PRDM16 with PGC 1α (71) and *PPARγ* (72) resulting in activation of the myogenic cascade (73) and BAT formation. Recent human studies have shown positive correlations between BAT volume and bone density (74–76). Nevertheless, *PPARy* remains the novel target because of its dual role in MSC-derived adipogenesis.as well as HSC-derived osteoclastogenesis (67). Studies of Beekman et al. (77) showed that *PPARγ* inhibitor, GW9962 has no direct impact on bone marrow adipose tissue (BMAT) in C3H/HeJ mice (77) suggesting that BMAT accumulation might be regulated by a different mechanism. In contrast, another study demonstrated upregulation of sphingosine-1-phosphate (S1P) by S1P lyase, mediated *PPARg* suppression resulting in enhanced bone formation (78). Similarly, Wnt cascade also plays a significant role in the initiation of adipogenesis in obese people (79, 80). Normally, Wnt ligands bind to one of the frizzled family receptors (FZD) and to a co-receptor low-density lipoprotein receptor-related protein (LRP) to activate β-catenin dependent pathway (canonical signaling) and subsequent bone formation (81, 82). Conversely, Wnt signal transduction seems to be redundant in both obesity (83) and T2DM (84). Previous studies showed a close relationship between upregulation of classical Wnt signaling and enhanced myogenesis and/or osteogenesis (85, 86). In humans, subcutaneous injection of Romosozumab which targets sclerostin (an inhibitor of the Wnt pathway), reduced the risk of vertebral and clinical fractures in women with postmenopausal osteoporosis and hence this drug was approved to treat osteoporosis (87). Thus, attractive therapeutic targets using Wnt-targets, acting on obesity associated genes such as secreted frizzled receptor(Srfp1) and Wnt inhibitory factor (WIF-1) acting on classical Wnt-β catenin pathway are undergoing pre-clinical and clinical trials (81).

### 1.4 Effect of T2DM and obesity on satellite cells and bone senescence

Sarcopenia which is defined as low muscle mass and function is common in the elderly and is associated with increased falls and fractures (88–93). It can accompany obesity in a significant number of older adults for a condition called sarcopenic obesity resulting in frailty (94). Exercise improved muscle strength and physical function in older adults (95–99) and mice (100). For instance, the Lifestyle Interventions and Independence for Elders (LIFE) study (101) in 424 sedentary older persons showed that engaging in moderate-intensity physical activity (combination of aerobic and resistance) intervention reduced the incidence of major mobility disability with an increase in the Short Physical Performance Battery (SPPB) (102). Similarly weight loss from lifestyle intervention by a combination diet and exercise improves physical function, and ameliorates frailty in obese older adults (103–105). In addition, these studies exercise added to diet resulted in amelioration of muscle and bone loss experienced by those who were on diet alone. Since obesity is a risk factor for T2DM, it is expected that a significant number of obese patients with T2DM also have sarcopenic obesity (106). It is likely that skeletal muscle mass and function relies on muscle progenitor cells cascade including satellite cells, interstitial progenitor cells and hence discovery of novel therapeutic targets to improve muscle mass and function are of utmost importance (107). Although the mechanism leading to impairment of muscle dysfunction in obesity remains unclear, the proinflammatory cytokines present in the muscles such *TNFα, IL-6* which are elevated in obesity has been found to be reduced by exercise (108).

Verpoorten et al., showed that cluster of differentiation (CD36) deficient mice although protected from diet-induced obesity, developed impaired satellite cell function and muscle regeneration (109). Apart from adipogenic and inflammatory markers, impairment in fatty acid uptake *via* CD36 can also affect bone integrity (110). Our recent studies showed that in patients with poorly-controlled T2DM had significantly higher circulating osteogenic precursor cells (COPs) compared to well-controlled diabetics. This could mean that COPs are markers of poor metabolic control or the possibility for uncontrolled hyperglycemia results in retardation of differentiation of COPs into mature osteoblasts (59). Studies from our lab also confirmed, that poor glycemic control over 1year is associated with poor bone microarchitecture and strength in men with T2DM (59, 111). On the other hand, alteration in crucial genes of myogenesis can promote development of osteoprogenitor cells. Studies from Hashimoto et al. (112), showed both primary and immortalized progenitor cells derived from muscle of healthy non-dystrophic woman expressed two osteoblastic specific bone proteins, alkaline phosphatase and Runt-related transcription factor 2 (*Runx2)* (112). Studies in knock-out mice (113) and other aging studies (114) also showed that Runx2 deficiency resulted in impairment in osteoblastogenesis and depletion for satellite cells. Thus, it is likely that satellite cells and its gene machinery, play significant role on mediating the process of bone repair and thus, can be used as strategy in treatment (115). The next section discusses on the targets to minimize/nullify the inflammatory oxidative stress and enhance osteogenesis.

## 2 Emerging therapeutic treatment in bone loss of obese and T2DM patients

Currently, there are numerous medications and therapeutic options for the treatment of osteoporosis but not for bone fragility in diabetic or obese patients in particular (116–120). Given this unmet need, understanding the pathways involved in bone disease in these patients will potentially lead to future strategies to prevent fractures.

### 2.1 Novel therapies -targeting bone formation

#### 2.1.1 Role of PRDM16 in adipo-myogenic shift and osteogenesis

**Figure 2:**
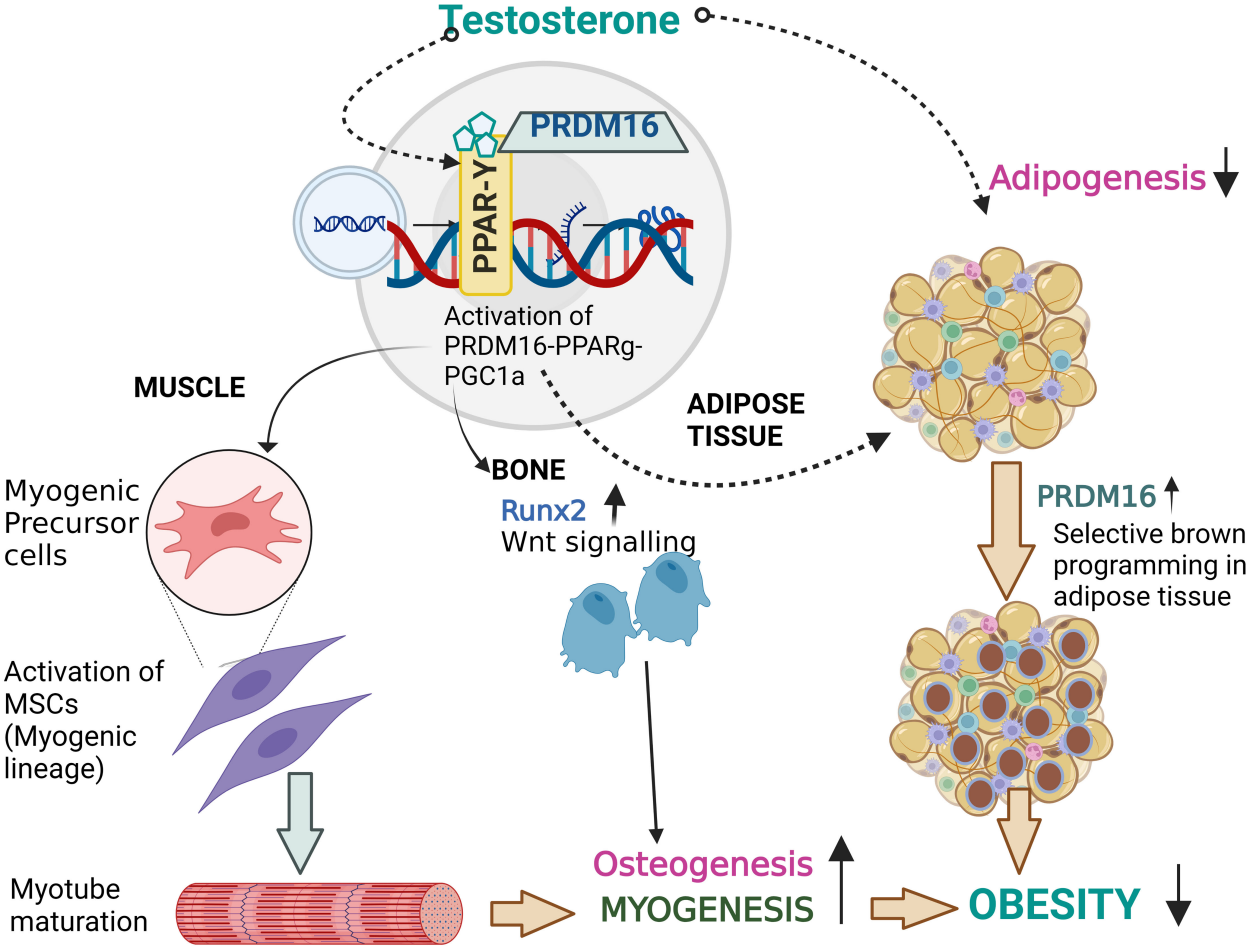
Proposed effect of T therapy in Obesity. Binding of testosterone might initiate the *PPARγ-Prdm16-Pgc1a* complex with retinoid X receptor (RXR) which not only activates the browning of adipose tissue by re-programming but may also activate the myogenic cascade and runx2 gene involved osteogenesis. Thus, T therapy with its activator *Prdm16* might be a novel therapeutic target and research on this transcriptional activator needs to be consider.

The novel therapeutic strategies that suppress bone marrow adipogenesis and bone resorption and enhanced bone formation deserve further research. Human PRDM16 located on chromosome 1p36 with 370kb, a zinc finger containing transcriptional regulator protein (121), was recently reported to interact with *PPARg* (122), *CEBPa* (123) and/or *Pgc-1a* (124) promote browning of fat. Additionally, *Prdm16* represses adipogenesis mediated through its association with C-terminal binding proteins (CtBP-1 and -2) suggesting that *PPARy* can act as bi-directional switch between adipogenesis and myogenesis through its interaction with multiple proteins (125). Apart from *Prdm16* and *PPARγ*, *Pgc1a* might act as co-activator and play critical role from adipogenic to myogenic shift. This was suggested by studies from Seo et al., showing reduction in obesity among mice fed a high-fat diet through suppression of adipogenesis by upregulation of *Prdm16*, *Pgc1a* and uncoupling protein 1 (UCP1) (126). Furthermore, Kaneda et al., found a synergistic association between *Prdm16* and Osteogenic *Runx2* gene in Mel1/*Prdm16*-deficient mice (127). They observed that BMP2 stimulated osteoblasts isolated from Mel1/Prdm16^+/-^ mice are highly stained with alizarin due to extensive calcification and enhanced expression of osteogenic markers such as osteopontin (OPN), OCN when compared to control mice (127). Thus, any ligand inducing a confirmational change in *PPARg* promoting the dissociation of transcriptional repressors and intake of co-activators (*Pgc1a*) leading to activation of the myogenic cascade (as described in **Figure 2**) along with promotion of the osteogenic *Runx2* gene might be a novel therapeutic targets. The research on these transcriptional activators needs to be investigated. In the next section, we explore the targets involved in myogenesis and osteogenesis and blocking of adipogenesis.

#### 2.1.2 Stem cell therapy

Obesity and T2DM enhance the recruitment of adipocyte precursors, resulting in fat deposition in the viscera, muscles, and other organs and bone fragility. Hence, it is critical to develop therapies to prevent adipocyte differentiation. Stem cell therapy remains an attractive candidate for tissue engineering (128). Adipose-derived mesenchymal stem cells (AD-MSCs) can exhibit various phenotypes of ecto and endodermal hematopoietic stem cells (HSCs) and mesodermal adipocytes, myocytes and osteocytes (129). Louwen et al. showed that human ASCs from obese patients had reduced capacity for osteogenic lineage differentiation (130, 131). Furthermore, Lee et al., reported that intra-articular injection of adipose derived AD-MSCs in patients with knee osteoarthritis, resulted in functional improvements for 6 months without major adverse effect (131). Thus, AD-MSC transplantation is feasible and can possibly be used to repair areas where osteoblastogenesis and subsequent endogenous bone formation is necessary (131, 132). This therapeutic potential of AD-MSCs depends on understanding the mechanism of differentiation capacity in the BM. In line with this, are over 1000 clinical trials registered with the Clinicaltrials.gov(http://www.Clinicaltrials.gov) which may demonstrate the clinical applications of AD-MSCs against bone fragility (133).

#### 2.1.3 Si RNA and other inhibitors

Targeted drug delivery strategies with reliable, efficient delivery remain crucial for cell-based therapy. Even though the delivery of siRNA to bone is challenging due to limited drug penetration and poor vascular perfusion, siRNAs (Short interfering RNA) play pivotal role than chemical-based studies (134), Previous studies targeting *Shn3* (adaptor protein Schnurri-3) gene silencing by genetically engineered BT-Exo-si*Shn3* novel MSC-derived exosome as carrier, resulted in osteogenesis along with blocking of Receptor activator of nuclear factor kappa-Β ligand/Dickkopf WNT Signaling Pathway Inhibitor 1 (RANKL/DKK-1), thereby inhibiting osteoclastogenesis in mouse MC3T3-E1 pre-osteoblast cell line (135). Liang et al, developed CH6 aptamer–functionalized lipid nanoparticles (LNPs), specifically targeting both rat and human osteoblasts, was found to promote bone formation (136). Due to high stability and non-immunogenicity aptamers, small single stranded oligonucleotides which can form 3D structure, are used in the ongoing clinical trials for their potential use as novel drug therapy targets for osteoporosis (137). Furthermore, in order to overcome the limitations of direct drug delivery, the combination of nanotechnology with bone target agents can provide more effective therapeutic approach in the near future (138).

#### 2.1.4 Testosterone therapy

Testosterone which is an old drug used for treatment of hypogonadism, has been found in recent years to have beneficial effects in both myogenesis and osteogenesis (139). T is well-known to improve BMD and bone quality in men (140–145). Various studies demonstrated that T therapy increased, levels of OCN (146, 147) and reduced levels of CTx (144, 145) (148) *In vitro* studies showed that 5α-dihydrotestosterone (a potent agonist of androgen receptor synthesized from T by the enzyme 5α-reductase) treatment of bone forming MC3T3-E1 cells not only enhanced osteoblast differentiation (149) but also downregulated bone resorption promoter RANKL (150) in human osteoblastic cells. Furthermore, testosterone administration increased the width of epiphyseal growth plate of growing rats directly (151, 152). Similarly, Chin et al, showed decreased trabecular bone volume and increased trabecular porosity in orchiectomized (ORX) rats when compared to sham-untreated (SH) group. Conversely, T treatment (7mg/kg) for 8weeks in ORX-TE group prevented these changes and decreased expression of RANKL significantly when compared to SH group (153).

Muscle function contributes in some measure to bone mass and testosterone increases muscle mass and function (154). Preclinical studies suggest a critical role of the adipogenic/myogenic/osteogenic switch on the observed effects of T therapy. Using mouse C3H 10T1/2 pluripotent cells, Singh et al, evaluated the effect of T treatment (0-300 nM) on the myogenic/adipogenic conversion by immunocytochemical staining for MyoD and PPARy (155). They found that T not only promotes commitment of SMSCs into the myogenic lineage but also inhibits adipogenic lineage. Apart from the myogenic machinery, Gao et al., further reported that osteoblast differentiation was activated by T therapy in MC3T3-E1 cells through ERK-1/2, activated *Runx2* pathway (156). Changes in body composition and bone density with T therapy from our lab and other investigators support the above findings from *in-vitro* and animal studies (146, 157, 158). Hence, we hypothesize that the reciprocal effect of T therapy on fat mass, lean mass and bone mass is due to the shift in lineage differentiation from adipogenesis to both myogenesis and osteogenesis. Thus, this concept provided a unifying mechanism for the observed effect of T in hypogonadal men. Roles of other gene machinery such as *Prdm16. Pgc1a* on the adipogenic/myogenic cascade need to be explored. We hypothesize that T therapy activates the trio cascade *PPARγ-Prdm16-Pgc1a* leading to initiation of the switch from adipogenesis to myogenesis along with promotion of osteogenesis (**Figure 2**) responsible for the observed positive effect on fat mass, lean mass and bone mass in hypogonadal men (146, 157, 158). The Endocrine Society has suggested the use of T to maintain or prevent loss of lean mass in men with HIV (159). Given the emergence of a substantial amount of data showing the positive effects of T on body composition and bone, it is possible that obesity may become one of the indications for T therapy.

## 3 Conclusion

Obesity and T2DM are increasing at an alarming rate worldwide. Despite the normal or better than normal BMD, both appear to be associated with increased fracture risk, most especially with T2DM. Though it is difficult to separate the skeletal effects of one from the other, there seems to be more data supporting the negative skeletal effects of T2DM than that of obesity, however, this is a complicated issue that needs further investigation. To date, there is no drug approved specifically to treat skeletal fragility in these patients. Since BMD cannot alone predict the risk of bone fragility, this review explores potential new methods or agents to promote the adipo-myogenic/osteogenic lineage shift which may include but not limited to targeting Prdm16, stem cell therapy, si-RNA inhibitors and repurposing of an old drug, testosterone in the general population of patients with obesity, T2DM or both. With further drug development, it is possible to prevent skeletal fragility and promote overall health in these patients.

## Author contributions

SB and RA-V, conceptualization, resources and analysis, writing, reviewing, and editing. The figures in this manuscript were created with biorender software. All authors contributed to the article and approved the submitted version.
